# A phase 3 trial evaluating panitumumab plus best supportive care *vs* best supportive care in chemorefractory wild-type *KRAS* or *RAS* metastatic colorectal cancer

**DOI:** 10.1038/bjc.2016.309

**Published:** 2016-10-13

**Authors:** Tae Won Kim, Anneli Elme, Zvonko Kusic, Joon Oh Park, Anghel Adrian Udrea, Sun Young Kim, Joong Bae Ahn, Ricardo Villalobos Valencia, Srinivasan Krishnan, Ante Bilic, Nebojsa Manojlovic, Jun Dong, Xuesong Guan, Catherine Lofton-Day, A Scott Jung, Eduard Vrdoljak

**Affiliations:** 1 Department of Oncology, Asan Medical Center, University of Ulsan, Seoul 138-736, South Korea; 2 North Estonia Medical Centre Foundation, Tallinn 13419, Estonia; 3 University Hospital Center ‘Sestre Milosrdnice', Zagreb 10000, Croatia; 4 Samsung Medical Center, Sungkyunkwan University School of Medicine, Seoul 135-710, South Korea; 5 MEDISPROF, Cluj-Napoca 400367, Romania; 6 National Cancer Center, Goyang-si, Gyeonggi-do 410-769, South Korea; 7 Yonsei University Health System Severance Hospital, Seoul 120-752, South Korea; 8 Centro Medico Nacional Siglo XXI, Mexico City 06725, Mexico; 9 Dr Rai Memorial Medical Centre, Chennai 600018, India; 10 Klinicka bolnica Sveti Duh, Zagreb 10000, Croatia; 11 Clinic for Gastroenterology and Hepatology of Military Medical Academy of Serbia, Belgrade 11000, Serbia; 12 Amgen Inc., Thousand Oaks, CA 91320, USA; 13 Department of Oncology, Center of Oncology, Clinical Hospital Center Split, Split 21000, Croatia

**Keywords:** gastrointestinal cancer, colorectal, phase 3 trial, panitumumab

## Abstract

**Background:**

We assessed the treatment effect of panitumumab plus best supportive care (BSC) *vs* BSC on overall survival (OS) in patients with chemorefractory wild-type *KRAS* exon 2 metastatic colorectal cancer (mCRC) and report the first prospective extended *RAS* analysis in a phase 3 trial.

**Methods:**

Patients with wild-type *KRAS* exon 2 mCRC were randomised 1 : 1 to panitumumab (6 mg kg^−1^ Q2W) plus BSC or BSC. On-study crossover was prohibited. *RAS* mutation status was determined by central laboratory testing. The primary endpoint was OS in wild-type *KRAS* exon 2 mCRC; OS in wild-type *RAS* mCRC (*KRAS* and *NRAS* exons 2, 3, and 4) was a secondary endpoint.

**Results:**

Three hundred seventy seven patients with wild-type *KRAS* exon 2 mCRC were randomised. Median OS was 10.0 months with panitumumab plus BSC *vs* 7.4 months with BSC (HR=0.73; 95% CI=0.57–0.93; *P*=0.0096). *RAS* ascertainment was 86%. In wild-type *RAS* mCRC, median OS for panitumumab plus BSC was 10.0 *vs* 6.9 months for BSC (HR=0.70; 95% CI=0.53–0.93; *P*=0.0135). Patients with *RAS* mutations did not benefit from panitumumab (OS HR=0.99; 95% CI=0.49–2.00). No new safety signals were observed.

**Conclusions:**

Panitumumab significantly improved OS in wild-type *KRAS* exon 2 mCRC. The effect was more pronounced in wild-type *RAS* mCRC, validating previous retrospective analyses.

Panitumumab, a fully human monoclonal antibody targeting the epidermal growth factor receptor (EGFR), is effective as monotherapy and in combination with chemotherapy for treatment of patients with metastatic colorectal cancer (mCRC) (Van Cutsem *et al*, 2007; Douillard *et al*, 2013; Poulin-Costello *et al*, 2013; Peeters *et al*, 2015). Signalling through the EGFR pathway initiates a number of signalling cascades that regulate cell proliferation (Mendelsohn and Baselga, 2006; Normanno *et al*, 2006). A key element of signal transduction through several of these cascades is activation of members of the RAS family of small GTP-binding proteins (Downward, 2003; Schubbert *et al*, 2007). Kirsten rat sarcoma-2 virus oncogene homologue (*KRAS*) is a member of the *RAS* gene family (Downward, 2003; Schubbert *et al*, 2007), and is frequently mutated in patients with CRC (Schubbert *et al*, 2007). Oncogenic mutations in *KRAS* are found most frequently in codons 12 and 13 of exon 2 and occur in ∼30–45% of CRC tumours (Lievre *et al*, 2006; Benvenuti *et al*, 2007; Di Fiore *et al*, 2007). These mutations compromise the GTP-binding domain, causing the protein to stall in the GTP-bound confirmation, thereby resulting in a constitutively active protein (Downward, 2003). Given the role of KRAS in EGFR signalling, it was hypothesised that these activating mutations in *KRAS* exon 2 may result in a lack of response to panitumumab. A retrospective analysis of the randomised, phase 3 20020408 study which evaluated panitumumab plus best supportive care (BSC) *vs* BSC alone found a significant improvement in patient outcomes in patients with wild-type *KRAS* exon 2 tumours compared with those who had mutant *KRAS* exon 2 tumours (Amado *et al*, 2008). These findings were subsequently confirmed by prospective analyses of the randomised, phase 3 PRIME and 20050181 studies, which evaluated panitumumab plus FOLFOX in the first-line setting or panitumumab plus FOLFIRI in the second-line setting, respectively (Douillard *et al*, 2010; Peeters *et al*, 2010).

More recent retrospective analyses have indicated that additional activating mutations in exons 3 and 4 of *KRAS* and in exons 2, 3, and 4 of *NRAS* (another member of the *RAS* family), are also negative predictors for anti-EGFR efficacy (Douillard *et al*, 2013; Schwartzberg *et al*, 2014; Bokemeyer *et al*, 2015; Peeters *et al*, 2015; Van Cutsem *et al*, 2015). These mutations have been identified in approximately 15% of wild-type *KRAS* exon 2 tumours (Douillard *et al*, 2013; Van Cutsem *et al*, 2015). Evaluation of outcomes in patients with wild-type *RAS* tumours (i.e., wild-type for *KRAS* and *NRAS,* exons 2, 3, and 4) by extended *RAS* analysis have shown numerically improved survival relative to patients with wild-type *KRAS* exon 2 tumours in the first-line (PRIME) (Douillard *et al*, 2013) and second-line (20050181) (Peeters *et al*, 2015) settings. In addition, outcomes for progression-free survival (PFS) and objective response rate (ORR) were more favourable in an extended *RAS* analysis from the 20020408 study (Patterson *et al*, 2013; Peeters *et al*, 2013). These analyses have helped guide treatment selection decisions to better refine patients who are likely to respond to anti-EGFR therapies and to exclude patients unlikely to respond, limiting potential exposure to toxicities. However, although these extended *RAS* analyses were rigorously conducted, they were retrospective in nature and were not prespecified endpoints in the respective study protocols at the time each study was initiated. Therefore, it is possible that potential sources of bias/confounding were not adequately mitigated. At present, *RAS* as a predictive biomarker for anti-EGFR therapies has yet to be validated in prospective, phase 3, randomised mCRC studies.

Although the primary objective of the phase 3, open-label, randomised 20100007 study (ClinicalTrials.gov, NCT01412957) was to evaluate the effect of panitumumab plus BSC *vs* BSC alone on overall survival (OS) in patients with chemotherapy-refractory wild-type *KRAS* exon 2 mCRC, a critical key secondary objective was to prospectively evaluate the treatment effect of panitumumab in patients with wild-type *RAS* mCRC. This analysis would provide definitive validation for *RAS* as a predictive biomarker for anti-EGFR therapies.

## Patients and methods

### Patient population

Eligible patients (⩾18 years) had histologically or cytologically confirmed metastatic adenocarcinoma of the colon/rectum, wild-type *KRAS* exon 2 (codons 12 and 13) tumour status confirmed by central laboratory (see below), Eastern Cooperative Oncology Group (ECOG) performance status ⩽2, ⩾1 measurable or non-measurable lesion per Response Evaluation Criteria In Solid tumours (RECIST) version 1.1 (Eisenhauer *et al*, 2009), had previously received a thymidylate synthase inhibitor (e.g., fluorouracil, capecitabine, raltitrexed or fluorouracil-uracil), and had clinical/radiological disease progression or toxicity on prior regimens for metastatic disease containing irinotecan and oxaliplatin. Relapse within 6 months after adjuvant chemotherapy was considered treatment failure for metastatic disease. Patients were excluded if they had symptomatic brain metastases requiring treatment; major surgery ⩽28 days before randomisation; clinically significant cardiovascular disease ⩽6 months before randomisation; previous anti-EGFR therapy (small-molecule or monoclonal antibody); magnesium below lower limit of normal; inadequate renal, hepatic or haematological function; antitumour therapy ⩽21 days before randomisation; and/or radiotherapy ⩽14 days before randomisation. Patients must have recovered from any acute chemotherapy/radiotherapy toxicities. It is important to note that at the time the trial opened for patient enrollment, a statistically significant OS benefit for panitumumab had not been observed nor had non-inferiority with other anti-EGFR therapies been established. Thus the trial was conceived and conducted under clinical equipoise. The study adhered to all country-specific regulatory requirements, the protocol was approved by an independent ethics committee at each of the study centres, and informed consent was obtained from all patients.

### Study design and treatment schedule

This was an open-label, randomised, phase 3 study conducted at 66 centres in 16 countries. Patients were randomly assigned 1 : 1 to the treatment arms using an interactive voice-response system to receive panitumumab 6 mg kg^−1^ intravenously on day 1 of each 14-day cycle plus BSC or BSC alone. Randomisation was stratified according to geographic region (Europe *vs* Asia *vs* rest of the world) and ECOG performance status (0 or 1 *vs* 2). BSC was defined as the best palliative care available, as judged appropriate by the investigator, consistent with institutional guidelines. BSC included antibiotics, analgesics, radiation for pain control (bone metastases only), corticosteroids, transfusions, psychotherapy, growth factors, palliative surgery or any other symptomatic therapy as clinically indicated. Patients were treated until disease progression, withdrawal of consent or panitumumab intolerance (panitumumab plus BSC arm only). Crossover from the BSC arm to panitumumab plus BSC was prohibited on-study.

### Assessments

Radiographic tumour assessments were performed at week 4 (+1 week), week 8 (±1 week) and every 8 weeks (±1 week) thereafter, until radiographic or clinical disease progression. Response was evaluated by investigators per RECIST version 1.1 (Eisenhauer *et al*, 2009). Patients were followed for survival for up to 24 months or until ∼250 deaths were observed, whichever occurred later. Patients had a safety follow-up visit 30–33 days after the last dose of panitumumab in the panitumumab plus BSC arm; and within 33 days of disease progression or the decision to end treatment in the BSC alone arm. Adverse events (AEs) occurring on-study were recorded and graded according to the Common Terminology Criteria for Adverse Events (CTCAE) version 3.0; skin- or nail-related AEs were graded using CTCAE version 3.0 with modifications.

### *RAS* mutational analysis

Three central laboratories screened patient tumour *KRAS* exon 2 status in formalin-fixed, paraffin-embedded tissue sections using validated *KRAS* assays that identified seven mutations in codons 12 and 13 to determine eligibility for panitumumab treatment (clinical trial assays based on primers from a DxS/Qiagen assay; Venlo, Netherlands). Extended *RAS* analysis was conducted in a single central laboratory (blinded to patients' treatment assignments/outcomes) on banked patient tumour specimens characterised as wild-type *KRAS* exon 2. Analyses of *KRAS* exon 3 (codons 59, 61) and exon 4 (codons 117, 146) and *NRAS* exon 2 (codons 12, 13), exon 3 (codons 59, 61) and exon 4 (codons 117, 146) were prespecified in the study protocol and mutation status determined by bidirectional Sanger sequencing before the primary analysis.

### Statistical analyses

The primary endpoint was OS (time from randomisation to death) for all patients with wild-type *KRAS* exon 2 mCRC. Secondary endpoints included PFS (time from randomisation to disease progression or death) and ORR (rate of either a complete response or partial response per RECIST version 1.1) (Eisenhauer *et al*, 2009) for patients with wild-type *KRAS* exon 2 status; OS, PFS and ORR for patients with wild-type *RAS* status; and safety. Assuming an HR (panitumumab plus BSC to BSC alone) of 0.66, to achieve 90% power for a 5% significance level test, a total of 250 OS events and a sample size of 350 patients were required. OS and PFS analyses included all patients randomised to treatment with wild-type *KRAS* exon 2 status (i.e., the intent-to-treat analysis set). Safety analyses included all randomised patients according to treatment received, which was the same as treatment randomised for all patients. Wild-type *RAS* analysis sets were similar and included patients with wild-type *KRAS* and *NRAS* exons 2, 3, and 4.

The primary analysis was planned when ∼250 deaths had occurred; no interim efficacy analysis was planned. Log-rank tests stratified by the randomisation factors (baseline ECOG performance status and region) were used to compare OS and PFS between treatment groups. Treatment effects on OS and PFS were estimated using stratified Cox proportional hazards models and the Kaplan–Meier method. Sequential testing based on a two-sided 5% significance level was used; if the OS treatment effect in the intent-to-treat population was significant, PFS in the intent-to-treat population would be tested. If PFS in the intent-to-treat population was significant, OS in the wild-type *RAS* population would be tested. If OS in the wild-type *RAS* population was significant, PFS in the wild-type *RAS* population would be tested. ORR was not formally tested.

*RAS* analyses were prospective in nature. Although treatment was not randomised within the wild-type *RAS* population, *RAS* analyses were specified as secondary objectives, *RAS* exons were defined in the protocol, a rigorous sequential testing methodology was prespecified, and the testing laboratories were blinded to both treatment assignment and clinical outcome.

## Results

### Patients

**Figure 1:**
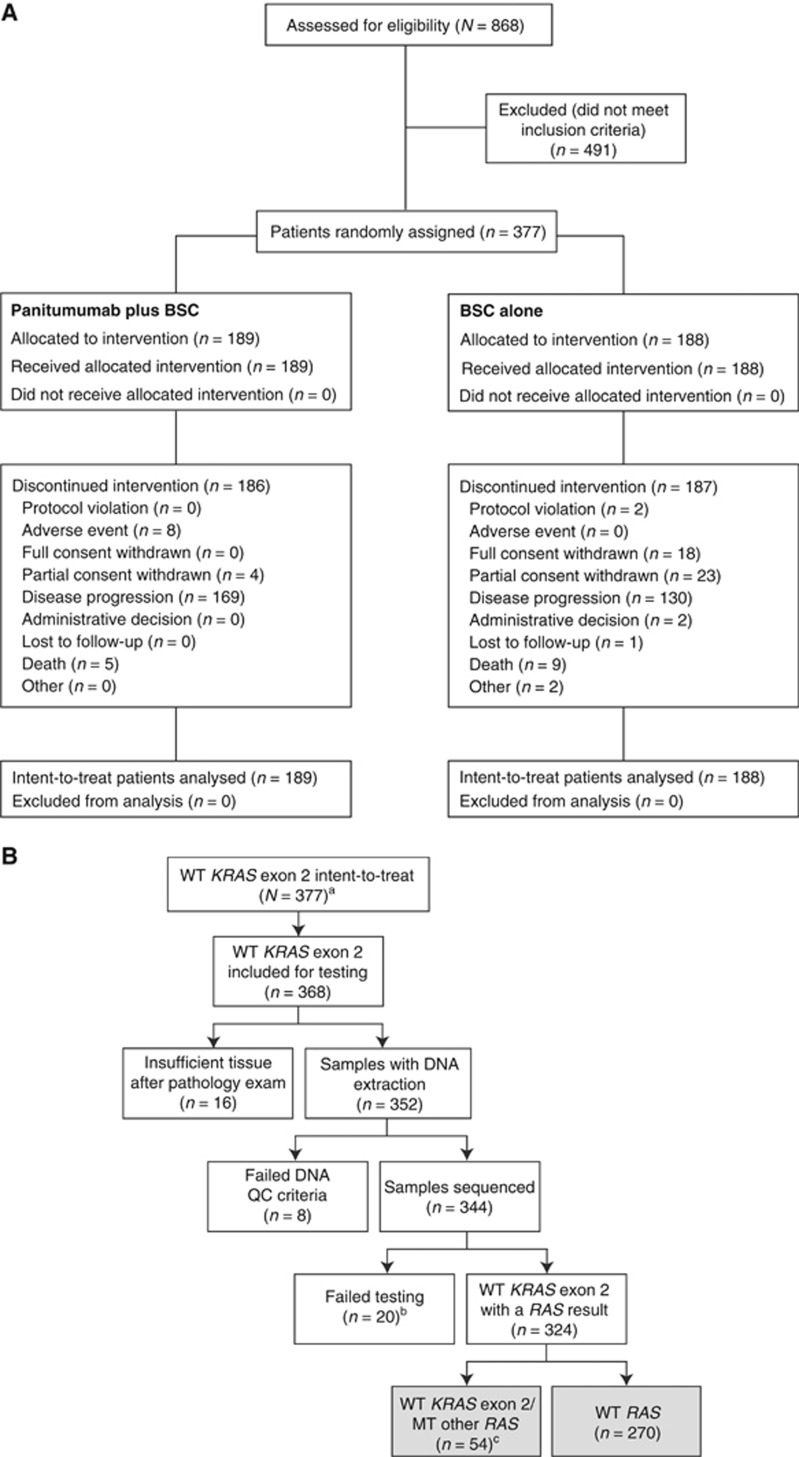
**CONSORT diagram (A) and *RAS* testing (B) for the 20100007 study.** ^a^*KRAS* exon 2 status was based on initial screening test results and wild-type for all 377 patients; mutation status of *KRAS* exon 3 and 4 and *NRAS* exons 2, 3, and 4 was determined by Sanger sequencing. ^b^Of 20 samples that failed, four were due to not meeting the analysis criteria and 16 had at least one *RAS* exon that failed testing and were wild-type in the other exons (see footnote c). ^c^If a sample had a *RAS* exon mutation (*KRAS* exon 3 or 4 or *NRAS* exons 2, 3 or 4) and one of the other exons failed testing, the sample was characterised as wild-type *KRAS* exon 2/mutant other *RAS*. BSC=best supportive care; QC=quality control; WT=wild-type.

Between November 2011 and July 2013, 377 patients with wild-type *KRAS* exon 2 mCRC were enrolled (panitumumab plus BSC, *n*=189; BSC alone, *n*=188; Figure 1A). Among these patients, 324 patients (86%) were evaluable for *RAS* analysis; 270 (83%) had tumours that were wild-type in exons 2–4 of *KRAS* and *NRAS* (i.e., wild-type *RAS*), and 54 (17%) had a mutation in *KRAS* exon 3 or 4 or *NRAS* exons 2, 3, or 4 (Figure 1B and Supplementary Table 1). Demographics and baseline characteristics were similar between arms in the wild-type *KRAS* exon 2, wild-type *RAS* and wild-type *KRAS* exon 2 but other *RAS* mutations populations (Table 1).

For patients who received panitumumab plus BSC, median (range) duration of treatment was 16.0 (2.0–80.0) weeks, and median (range) number of infusions was 8.0 (1–40). For patients who received BSC alone, median (range) duration of treatment was 5.1 (0–56.4) weeks. Median follow-up time was 41.0 and 25.5 weeks for panitumumab plus BSC and BSC alone, respectively. At the time of analysis, 99% of patients had discontinued study therapy, most frequently due to disease progression (79% Figure 1A). After disease progression, 29% of patients in the panitumumab plus BSC arm and 34% of patients in the BSC alone arm received additional antitumour therapy including chemotherapy (panitumumab plus BSC, 26% BSC alone, 21%), anti-EGFR therapy (1% 7%) and bevacizumab (2% 5%).

### Efficacy

**Figure 2:**
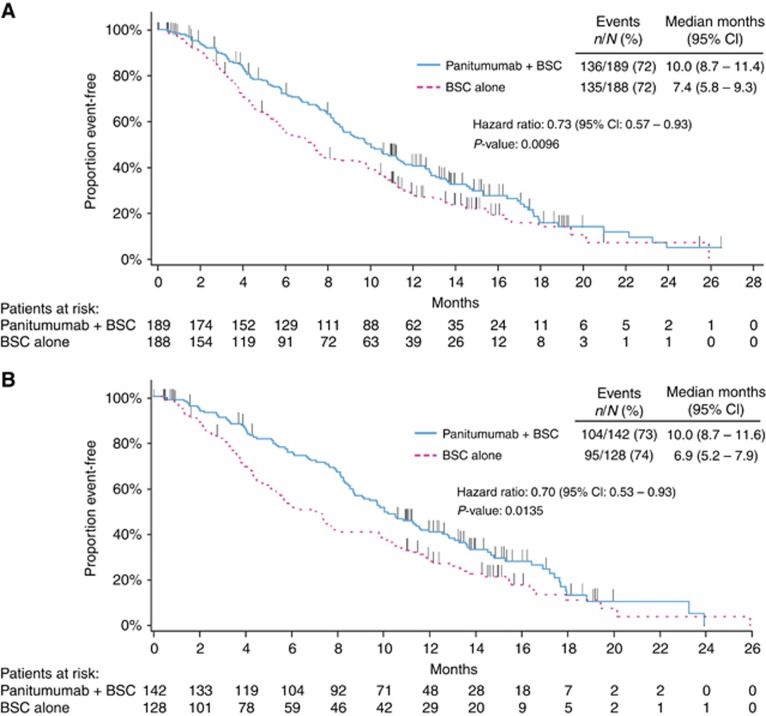
**Overall survival in (A) the wild-type *KRAS* exon 2 group and (B) in the extended wild-type *RAS* subgroup.** BSC=best supportive care.

Panitumumab plus BSC significantly improved OS compared with BSC alone. For patients with wild-type *KRAS* exon 2 tumours, median (95% CI) OS was 10.0 (8.7–11.4) months in the panitumumab plus BSC arm *vs* 7.4 (5.8–9.3) months in the BSC alone arm (HR=0.73; 95% CI=0.57–0.93; *P*=0.0096; Figure 2A, Table 2). In OS subgroups defined by baseline characteristics, HRs consistently favoured panitumumab plus BSC over BSC alone including subgroups by ECOG status (0/1 or 2), metastatic sites (1, 2 or ⩾3) and liver limited disease (Supplementary Figure 1A).

**Figure 3:**
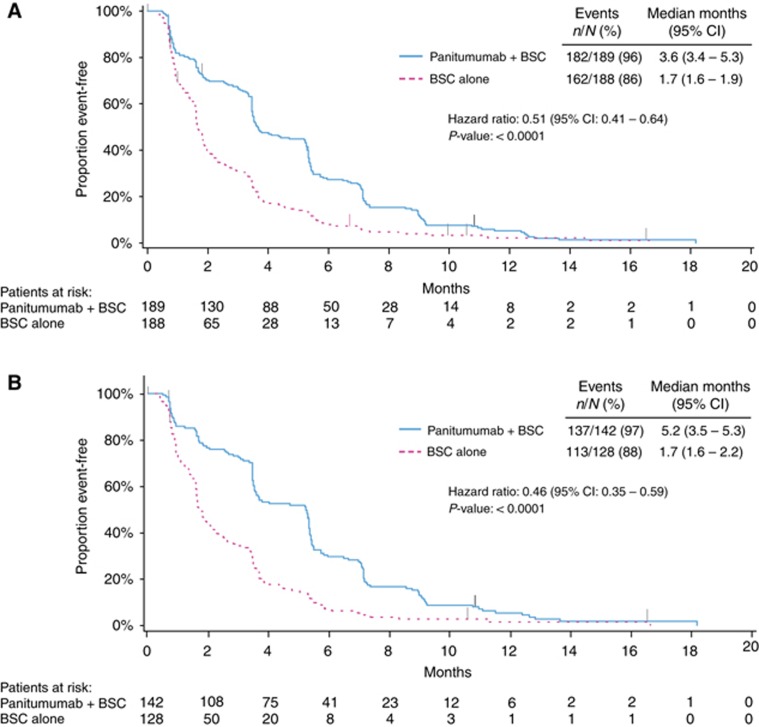
**Progression-free survival in (A) the wild-type *KRAS* exon 2 group and (B) in the extended wild-type *RAS* subgroup.** BSC=best supportive care.

PFS was also improved among patients in the panitumumab arm *vs* those in the BSC alone arm. Median (95% CI) PFS was 3.6 (3.4–5.3) months for panitumumab plus BSC *vs* 1.7 (1.6–1.9) months for BSC alone (HR=0.51; 95% CI=0.41–0.64; *P*<0.0001; Figure 3A, Table 2). The ORR (95% CI) for panitumumab plus BSC was 27.0% (20.8–33.9) *vs* 1.6% (0.3–4.6) for BSC alone (odds ratio (OR)=24.9; 95% CI=7.5–123.8; *P*<0.0001; Table 2).

As in the wild-type *KRAS* exon 2 population, treatment with panitumumab plus BSC improved OS *vs* BSC alone for patients with wild-type *RAS* tumours (median (95% CI): 10.0 (8.7–11.6) months *vs* 6.9 (5.2–7.9) months; HR=0.70; 95% CI=0.53–0.93; *P*=0.0135; Figure 2B, Table 2). In OS subgroups defined by baseline characteristics, results also favoured panitumumab plus BSC over BSC alone (Supplementary Figure 1B). PFS was improved for panitumumab plus BSC (median (95% CI): 5.2 (3.5–5.3) months) *vs* BSC alone (1.7 (1.6–2.2) months; HR=0.46; 95% CI=0.35–0.59; *P*<0.0001; Figure 3B, Table 2). In addition, ORR (95% CI) for panitumumab plus BSC was 31.0% (23.5–39.3) *vs* 2.3% (0.5–6.7) for BSC alone (OR=20.0; 95% CI=5.9–101.6; *P*<0.0001; Table 2).

Patients with wild-type *KRAS* exon 2 tumours but with other *RAS* mutations did not benefit from panitumumab therapy. In this group, the HRs (95% CI) for OS and PFS for panitumumab plus BSC *vs* BSC alone were 0.99 (0.49–2.00; Supplementary Figure 2A) and 1.03 (0.56–1.90; Supplementary Figure 2B), respectively. Complete or partial responses were not achieved by either group; stable disease was achieved by 42% for panitumumab plus BSC *vs* 21% for BSC alone (Supplementary Table 2).

### Safety

In the wild-type *KRAS* exon 2 population, 97% of those who received panitumumab plus BSC and 61% of those who received BSC alone experienced an AE of any grade (Table 3). The incidences of grades 3 and 4 AEs were 37% and 9%, respectively, for panitumumab plus BSC and 15% and 3% for BSC alone (Table 3). Eight patients (4%) in the panitumumab plus BSC arm discontinued treatment because of AEs *vs* 0 patients (0%) in the BSC alone arm. Fatal AEs occurred in 8 (4%) patients who received panitumumab plus BSC (seven due to disease progression and one to gastrointestinal necrosis) *vs* 15 (8%) patients who received BSC alone (nine due to disease progression and one each to bone marrow toxicity, congestive cardiac failure, abnormal hepatic function, lower gastrointestinal haemorrhage, respiratory distress and sepsis).

The incidence of infusion reactions was 1% in the panitumumab plus BSC arm. AEs of any grade with a ⩾5% difference between arms (panitumumab plus BSC, BSC alone) included rash (39%, 1%), dermatitis acneiform (29%, 0%) and hypomagnesemia (28%, 1% Supplementary Table 3, online only). Incidences of grade 3/4 hypomagnesemia, skin rash, and dermatitis acneiform were 6%, 6%, and 6%, respectively, for panitumumab plus BSC, and 1%, 1%, and 0% for BSC alone (Supplementary Table 3). Similar incidences were observed for the wild-type *RAS* and the wild-type *KRAS* exon 2 but mutant other *RAS* populations (Supplementary Tables 4 and 5).

## Discussion

This was the first randomised, phase 3 study to demonstrate a significant OS benefit with panitumumab monotherapy *vs* BSC in patients with wild-type *KRAS* exon 2 mCRC. In the 20020408 study of panitumumab plus BSC *vs* BSC alone, OS benefit was not shown, likely because 76% of patients in the BSC arm opted to cross over and receive panitumumab as allowed in the study protocol (Van Cutsem *et al*, 2007). Retrospective analyses suggested OS may have been improved if crossover had not occurred (Poulin-Costello *et al*, 2013). Although caution is warranted when making cross-trial comparisons, median OS in the panitumumab arm in the wild-type *KRAS* population in this study (10.0 months, 95% CI=8.7–11.4) was moderately longer relative to that in the wild-type *KRAS* population in the 20020408 study (8.1 months) but was consistent with that reported in the ASPECCT study (panitumumab, 10.4 months, 95% CI=9.4–11.6 months; cetuximab, 10.0 months, 95% CI=9.3–11.0 months; HR=0.97, 95% CI=0.84–1.11) (Price *et al*, 2014) and in the cetuximab CO.17 study (9.5 months) (Karapetis *et al*, 2008). The OS HR in this study was 0.73 and was higher than the anticipated HR of 0.66. The assumed OS HR was based on results from the CO.17 study where median OS in the control arm was 4.8 months. The longer median OS for the BSC arm reported in this study (7.4 months) likely impacted the HR and suggests that outcomes with BSC have improved over time. The longer OS time in the BSC arm observed in this study, compared with the 20020408 study, may have resulted from the more extensive use of post-treatment antitumour therapy after disease progression (34% of patients in the BSC arm in 20100007 received post-progression therapy). In the wild-type *KRAS* exon 2 population, median PFS in the panitumumab arm was also longer than in the BSC arm (3.6 months, 95% CI=3.4–5.3 months *vs* 1.7 months; 95% CI=1.6–1.9 months). Notably, PFS in the BSC arm was consistent between the 20020408 and 20100007 populations, providing further evidence that the differences in OS times between the studies may have been due to post-progression therapy. No new safety signals were observed in this study. Events occurring more frequently among patients who received panitumumab were consistent with previous studies (Douillard *et al*, 2010; Price *et al*, 2014; Peeters *et al*, 2015), and the rate of infusion reactions with panitumumab therapy was low (1%).

This was also the first phase 3 study to demonstrate significant improvements in OS, PFS and ORR with an anti-EGFR agent in a prospectively defined chemorefractory wild-type *RAS* population (although, as noted, treatment was not randomised within the wild-type *RAS* population). These results confirm and extend those from previous retrospective analyses evaluating extended *RAS* analysis in mCRC (Douillard *et al*, 2013; Peeters *et al*, 2013; Bokemeyer *et al*, 2015; Peeters *et al*, 2015; Van Cutsem *et al*, 2015). In the 20020408 study of panitumumab plus BSC *vs* BSC alone, PFS was significantly improved in patients with wild-type *KRAS* exon 2 tumours (HR=0.45; 95% CI=0.34–0.59) (Amado *et al*, 2008), and this treatment effect was greater in retrospective analysis in the wild-type *RAS* population (HR=0.39; 95% CI=0.27–0.56) (Peeters *et al*, 2013). In the 20100007 study, improvements in PFS were also seen in both the wild-type *KRAS* exon 2 population (HR=0.51; 95% CI=0.41–0.64) and the wild-type *RAS* population (HR=0.46; 95% CI=0.34–0.59). The improvement in outcomes in the wild-type *RAS* population *vs* the wild-type *KRAS* exon 2 population is also consistent with that seen in extended *RAS* analysis of the PRIME study in the first-line setting (Douillard *et al*, 2013) and the 20050181 study in the second-line setting (Peeters *et al*, 2015), indicating the benefit observed in patients with tumours without activating *RAS* mutations is true across all lines of therapy and for combination treatment as well as monotherapy. In both the 20020408 study and in this study, patients who were wild-type for *KRAS* exon 2 but had other *RAS* mutations (i.e., in either *KRAS* exons 3 or 4 or *NRAS* exons 2, 3 or 4) were unlikely to benefit from panitumumab. This lack of benefit is consistent with results from other monotherapy studies and studies evaluating combination therapy (Bokemeyer *et al*, 2009; Douillard *et al*, 2013; Peeters *et al*, 2015; Van Cutsem *et al*, 2015).

The consistency between outcomes in the 20020408 study and the 20100007 study demonstrates the value of rigorously conducted retrospective analyses. The retrospective extended *RAS* analyses of the randomised, phase 3 20020408, PRIME and 20050181 studies were all conducted following stringent analysis procedures (Douillard *et al*, 2013; Peeters *et al*, 2013; Peeters *et al*, 2015), providing a rigorous framework for analysis that mirrored the procedures followed for the prospective extended *RAS* analysis of the 20100007 study. First, in both the retrospective and prospective analyses, tissue samples were collected with appropriate informed consent before patients were randomised to treatment arms. Second, the extended *RAS* analyses conducted for these studies were predefined: the statistical analysis plans were finalised before patient biomarker status information became available. Third, the testing laboratories were all blinded to both treatment assignment and clinical outcome. Finally, these retrospective analyses used data from large, randomised controlled phase 3 trials, and the high *RAS* ascertainment rates for these studies minimised the potential for ascertainment bias due to missing data on tumour *RAS* status. *RAS* ascertainment rates ranged from 75% in the 20020408 study (Patterson *et al*, 2013), 90% in the PRIME study (Douillard *et al*, 2013), 85% in the 20050181 study (Peeters *et al*, 2015), to 86% in this study. In the *RAS*-evaluable populations, reported rates of *RAS* mutations (*KRAS* exons 3 or 4 or *NRAS* exons 2, 3 or 4) found in the wild-type *KRAS* exon 2 population were between 15 and 20% (Douillard *et al*, 2013; Peeters *et al*, 2015), and the 17% frequency observed here in the 20100007 study was consistent with this range. The high ascertainment rate, coupled with the large informative patient populations in the phase 3 trials, provided numerically large patient subgroups for the expanded analyses of activating *RAS* mutations, thus limiting the potential for influence of outliers on outcomes. Given the rigorous nature of these analyses and the consistency of improvement in outcomes in the wild-type *RAS* population *vs* the wild-type *KRAS* exon 2 population in each study, the studies as a whole provide reassurance that the better predictive value of extended *RAS* analysis is a genuine finding and not an experimental artefact. Just as the predictive value of *KRAS* exon 2 mutations was identified in retrospective analysis of the 20020408 study (Amado *et al*, 2008) and confirmed by prospective analysis of the PRIME and 20050181 studies (Douillard *et al*, 2010; Peeters *et al*, 2010), similarly, the predictive value of extended *RAS* analysis was identified in retrospective analyses from the PRIME and 20050181 studies (Douillard *et al*, 2013; Peeters *et al*, 2015) and confirmed by the prospective extended *RAS* analysis in the 20100007 study. Taken together, these findings indicate that when retrospective analyses are conducted within a predefined rigorous, controlled methodological framework they can serve as an important and valid method for identifying predictive biomarkers, particularly *in situations* where it may be challenging to conduct a large prospective trial

Although the results from the 20100007 study validate those seen in previous studies, the study did have limitations that may have influenced outcomes. First, although on-study crossover was prohibited, the evaluation of OS may have been confounded by subsequent therapy use. Patients in the BSC alone arm were more likely to receive anti-EGFR therapy or bevacizumab after progression; however, this imbalance would be anticipated to reduce the magnitude of the treatment effect, yet statistically significant improvements were still seen. Second, the extended *RAS* analysis was performed after randomisation and before the planned primary analysis, and thus the treatment assignment was not randomised within the wild-type *RAS* subgroup. Although this could potentially have confounded the study results, it must be noted that the distribution of identified *RAS* activating mutations (*KRAS* exons 3 or 4 or *NRAS* exons 2, 3 or 4) was similar between treatment arms and the high ascertainment rate provided a lower probability of ascertainment bias.

The results from the prospective 20100007 trial indicate panitumumab therapy significantly improved OS and PFS, and numerically improved ORR in patients with chemotherapy-refractory wild-type *KRAS* exon 2 and wild-type *RAS* mCRC *vs* BSC alone. *RAS* analysis from the 20100007 study definitively confirms the negative predictive value of activating *RAS* mutations for response in patients with mCRC receiving panitumumab monotherapy. Moreover, the concordance observed between the prospective 20100007 study and the retrospective 20020408, PRIME and 20050181 analyses provides strong supporting evidence for the use of prespecified high ascertainment retrospective analyses to effectively identify treatment predictive biomarkers for mCRC outcomes.

## Figures and Tables

**Table 1:** Baseline demographics and disease characteristics

	**Wild-type** ***KRAS*** **exon 2**		**Wild-type *RAS***		**Wild-type** ***KRAS*** **exon 2/mutant other** ***RAS*** **exons**	
	**Panitumumab plus BSC (*****n*****=189)**	**BSC alone (*****n*****=188)**	**Panitumumab plus BSC (*****n*****=142)**	**BSC alone (*****n*****=128)**	**Panitumumab plus BSC (*****n*****=26)**	**BSC alone (*****n*****=28)**
Age, years, median (range)	62.0 (30–82)	60.0 (19–79)	62.0 (30–82)	59.5 (19–79)	62.0 (32–82)	62.5 (49–75)
Men, *n* (%)	107 (56.6)	109 (58.0)	80 (56.3)	77 (60.2)	11 (42.3)	12 (42.9)
Race, white, *n* (%)	107 (56.6)	102 (54.3)	82 (57.7)	71 (55.5)	14 (53.8)	19 (67.9)
Geographic region						
Europe	86 (45.5)	85 (45.2)	66 (46.5)	55 (43.0)	9 (34.6)	18 (64.3)
Asia	80 (42.3)	82 (43.6)	59 (41.5)	55 (43.0)	12 (46.2)	8 (28.6)
Rest of the world	23 (12.2)	21 (11.2)	17 (12.0)	18 (14.1)	5 (19.2)	2 (7.1)
ECOG status, *n* (%)						
0	71 (37.6)	65 (34.6)	54 (38.0)	42 (32.8)	5 (19.2)	10 (35.7)
1	100 (52.9)	107 (56.9)	73 (51.4)	75 (58.6)	18 (69.2)	16 (57.1)
2	18 (9.5)	16 (8.5)	15 (10.6)	11 (8.6)	3 (11.5)	2 (7.1)
Primary tumour diagnosis, *n* (%)						
Colon	108 (57.1)	106 (56.4)	88 (62.0)	72 (56.3)	9 (34.6)	17 (60.7)
Rectum	81 (42.9)	81 (43.1)	54 (38.0)	55 (43.0)	17 (65.4)	11 (39.3)
Missing	0 (0.0)	1 (0.5)	0 (0.0)	1 (0.8)	0 (0.0)	0 (0.0)
Number of metastatic sites, *n* (%)						
1	42 (22.2)	37 (19.7)	33 (23.2)	23 (18.0)	4 (15.4)	4 (14.3)
2	63 (33.3)	68 (36.2)	50 (35.2)	47 (36.7)	9 (34.6)	12 (42.9)
⩾3	84 (44.4)	83 (44.1)	59 (41.5)	58 (45.3)	13 (50.0)	12 (42.9)
Liver-only metastatic disease, *n* (%)	18 (9.5)	20 (10.6)	17 (12.0)	13 (10.2)	0 (0.0)	2 (7.1)
Prior bevacizumab treatment, *n* (%)						
Yes	63 (33.3)	57 (30.3)	48 (33.8)	34 (26.6)	6 (23.1)	15 (53.6)
No	126 (66.7)	131 (69.7)	94 (66.2)	94 (73.4)	20 (76.9)	13 (46.4)

Abbreviations: BSC=best supportive care; ECOG=Eastern Cooperative Oncology Group.

**Table 2:** Efficacy results

	**Wild-type** ***KRAS*** **exon 2**		**Wild-type *RAS***	
	**Panitumumab plus BSC (*****n*****=189)**	**BSC alone (*****n*****=188)**	**Panitumumab plus BSC (*****n*****=142)**	**BSC alone (*****n*****=128)**
**Overall survival, events, %**	136 (72.0)	135 (71.8)	104 (73.2)	95 (74.2)
Median (95% CI), months	10.0 (8.7–11.4)	7.4 (5.8–9.3)	10.0 (8.7–11.6)	6.9 (5.2–7.9)
** Hazard ratio (95% CI)**	0.73 (0.57–0.93)		0.70 (0.53–0.93)	
*P*-value	0.0096		0.0135	
**Progression-free survival, events, %**	182 (96.3)	162 (86.2)	137 (96.5)	113 (88.3)
Median (95% CI), months	3.6 (3.4–5.3)	1.7 (1.6–1.9)	5.2 (3.5–5.3)	1.7 (1.6–2.2)
Hazard ratio (95% CI)	0.51 (0.41–0.64)		0.46 (0.35–0.59)	
*P*-value	<0.0001		<0.0001	
**Patients receiving subsequent therapy, *n* (%)**				
Chemotherapy	50 (26.5)	40 (21.3)	45 (31.7)	28 (21.9)
Anti-EGFR therapy	2 (1.1)	14 (7.4)	0 (0.0)	11 (8.6)
Bevacizumab	3 (1.6)	10 (5.3)	3 (2.1)	7 (5.5)
**Objective response, *N***	189	188	142	128
Complete response, *n* (%)	0 (0.0)	0 (0.0)	0 (0.0)	0 (0.0)
Partial response, *n* (%)	51 (27.0)	3 (1.6)	44 (31.0)	3 (2.3)
Stable disease, *n* (%)	79 (41.8)	38 (20.2)	62 (43.7)	26 (20.3)
Disease progression, *n* (%)	53 (28.0)	95 (50.5)	31 (21.8)	62 (48.4)
Unevaluable/not done, *n* (%)	6 (3.2)	52 (27.7)	5 (3.5)	37 (29.0)
**Objective response rate, % (95% CI)**	27.0 (20.8–33.9)	1.6 (0.3–4.6)	31.0 (23.5–39.3)	2.3 (0.5–6.7)
Odds ratio (95% CI)	24.89 (7.47–123.77)		20.00 (5.89–101.6)	
*P*-value	<0.0001		<0.0001	

Abbreviations: BSC=best supportive care; CI=confidence interval; EGFR=epidermal growth factor receptor.

**Table 3:** Summary of adverse events

	**Wild-type *KRAS* exon 2**		**Wild-type *RAS***	
	**Panitumumab plus BSC (*n*=189)**	**BSC alone (*n*=188)**	**Panitumumab plus BSC (*n*=142)**	**BSC alone (*n*=128)**
**Patients with any adverse event, *n* (%)**	184 (97.4)	115 (61.2)	138 (97.2)	79 (61.7)
Worst grade of 3	70 (37.0)	29 (15.4)	55 (38.7)	20 (15.6)
Worst grade of 4	17 (9.0)	5 (2.7)	10 (7.0)	4 (3.1)
Worst grade of 5	8 (4.2)	15 (8.0)	6 (4.2)	10 (7.8)
Any serious	48 (25.4)	37 (19.7)	30 (21.1)	26 (20.3)
**Leading to permanent discontinuation of any study drug**	20 (10.6)	—	11 (7.7)	—
Not serious	14 (7.4)	—	7 (4.9)	—
Serious	6 (3.2)	—	4 (2.8)	—

Abbreviation: BSC=best supportive care.
