# Supplementary figures and images for: A phase 3 trial evaluating panitumumab plus best supportive care vs best supportive care in chemorefractory wild-type KRAS or RAS metastatic colorectal cancer
Source: Br J Cancer. 2016 Oct 13;115(10):1206–14. doi: 10.1038/bjc.2016.309 (PMC5104888; doi:10.1038/bjc.2016.309)

A

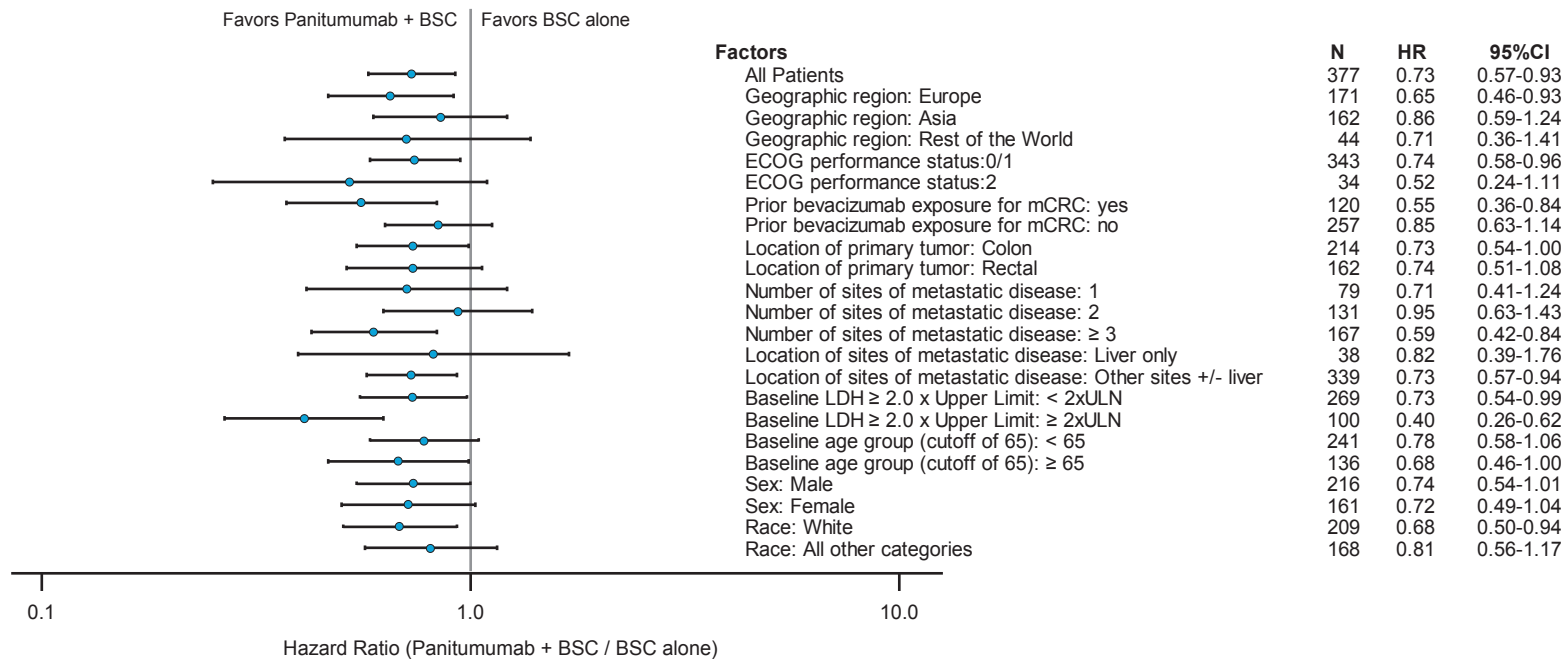

B

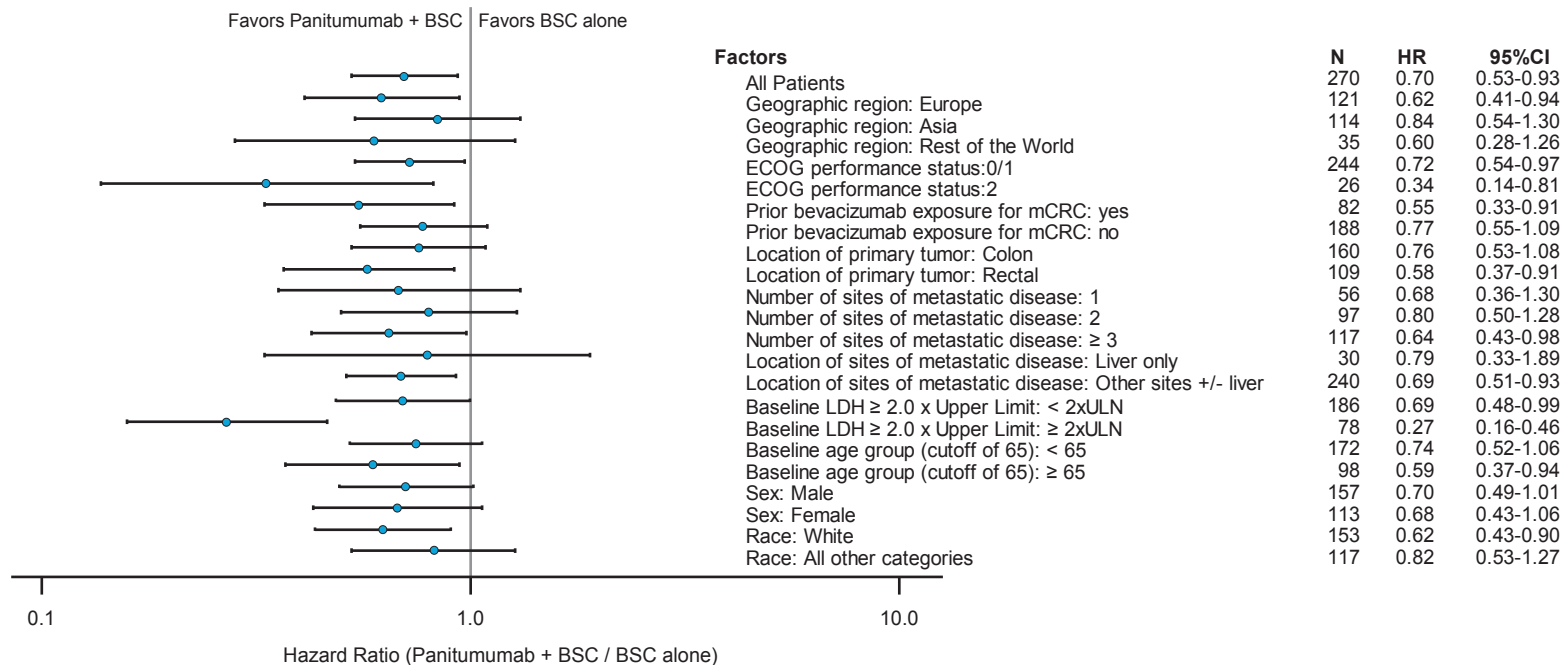

Supplement: Supplementary Figure 1 [file bjc2016309x1.pdf]

**A**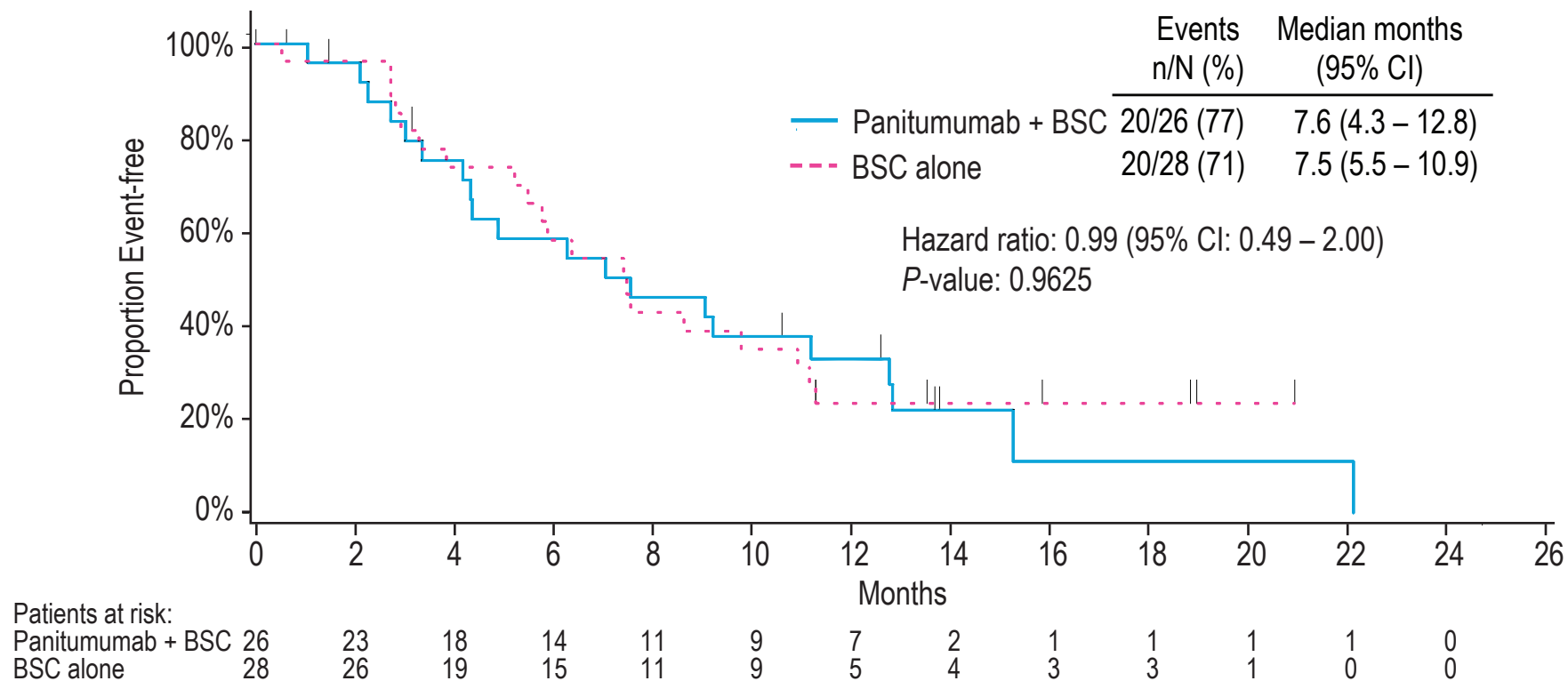**B**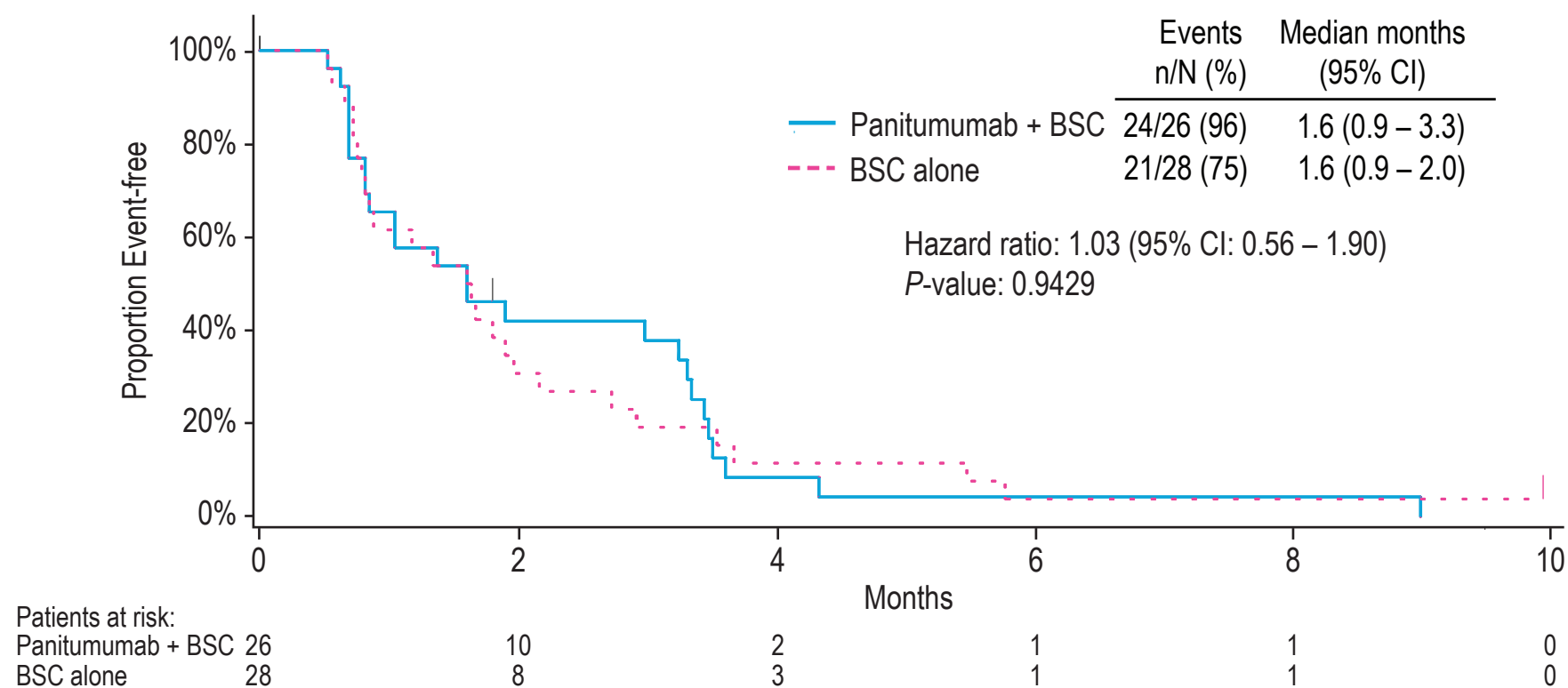

Supplement: Supplementary Figure 2 [file bjc2016309x2.pdf]
